# Corneal epithelial differentiation of human pluripotent stem cells generates ABCB5^+^ and ∆Np63α^+^ cells with limbal cell characteristics and high wound healing capacity

**DOI:** 10.1186/s13287-021-02673-3

**Published:** 2021-12-20

**Authors:** Meri Vattulainen, Tanja Ilmarinen, Taina Viheriälä, Vilma Jokinen, Heli Skottman

**Affiliations:** Faculty of Medicine and Health Technology, Tampere University, Arvo Ylpön katu 34, 33520 Tampere, Finland

**Keywords:** Human pluripotent stem cells, Stem cell differentiation, Limbal stem cells, Limbal stem cell deficiency, ABCG2, ABCB5, Wound healing, Calcium signaling

## Abstract

**Background:**

Differentiation of functional limbal stem cells (LSCs) from human pluripotent stem cells (hPSCs) is an important objective which can provide novel treatment solutions for patients suffering from limbal stem cell deficiency (LSCD). Yet, further characterization is needed to better evaluate their immunogenicity and regenerative potential before clinical applications.

**Methods:**

Human PSCs were differentiated towards corneal fate and cryopreserved using a clinically applicable protocol. Resulting hPSC-LSC populations were examined at days 10–11 and 24–25 during differentiation as well as at passage 1 post-thaw. Expression of cornea-associated markers including PAX6, ABCG2, ∆Np63α, CK15, CK14, CK12 and ABCB5 as well as human leukocyte antigens (HLAs) was analyzed using immunofluorescence and flow cytometry. Wound healing properties of the post-thaw hPSC-LSCs were assessed via calcium imaging and scratch assay. Human and porcine tissue-derived cultured LSCs were used as controls for marker expression analysis and scratch assays at passage 1.

**Results:**

The day 24–25 and post-thaw hPSC-LSCs displayed a similar marker profile with the tissue-derived LSCs, showing abundant expression of PAX6, ∆Np63α, CK15, CK14 and ABCB5 and low expression of ABCG2. In contrast, day 10–11 hPSC-LSCs had lower expression of ABCB5 and ∆Np63α, but high expression of ABCG2. A small portion of the day 10–11 cells coexpressed ABCG2 and ABCB5. The expression of class I HLAs increased during hPSC-LSCs differentiation and was uniform in post-thaw hPSC-LSCs, however the intensity was lower in comparison to tissue-derived LSCs. The calcium imaging revealed that the post-thaw hPSC-LSCs generated a robust response towards epithelial wound healing signaling mediator ATP. Further, scratch assay revealed that post-thaw hPSC-LSCs had higher wound healing capacity in comparison to tissue-derived LSCs.

**Conclusions:**

Clinically relevant LSC-like cells can be efficiently differentiated from hPSCs. The post-thaw hPSC-LSCs possess functional potency in calcium responses towards injury associated signals and in wound closure. The developmental trajectory observed during hPSC-LSC differentiation, giving rise to ABCG2^+^ population and further to ABCB5^+^ and ∆Np63α^+^ cells with limbal characteristics, indicates hPSC-derived cells can be utilized as a valuable cell source for the treatment of patients afflicted corneal blindness due to LSCD.

**Graphical Abstract:**

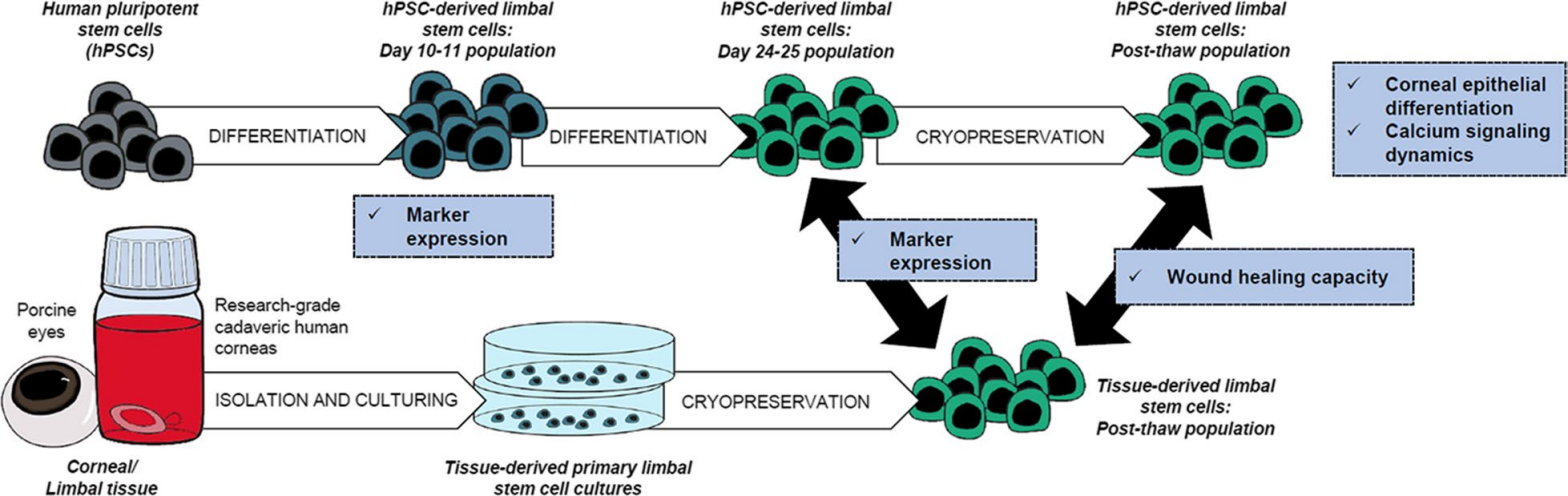

**Supplementary Information:**

The online version contains supplementary material available at 10.1186/s13287-021-02673-3.

## Background

Corneal limbal stem cells (LSCs) reside in the limbus, the anatomically and microenvironmentally specialized zone separating peripheral cornea from the adjacent conjunctiva. LSCs play an essential role in maintaining homeostatic renewal and transparency of the multilayered corneal epithelium (CE) [1]. Upon injury, activation of the quiescent LSCs facilitates corneal epithelial wound healing [2]. LSC loss or dysfunction leads to severe visual impairment due to emanating epithelial defects, conjunctival ingrowth, neovascularization, and opacification of the normally clear and avascular corneal surface. This condition, also known as limbal stem cell deficiency (LSCD), can be caused by chemical or thermal burns, and inflammatory or genetic eye disorders [3]. Despite multiple proposed strategies for the treatment of LSCD [4], transplantation of autologous or allogeneic LSCs remains the only curative option [5–7]. The currently preferred approach to unilateral LSCD involves transplantation of either a limbal autograft obtained by biopsy from the healthy contralateral eye, i.e., simple epithelial transplantation (SLET), or of ex vivo cultivated autologous LSCs (CLET). Treatment of bilateral LSCD involves transplantation of LSCs obtained from an allogeneic donor. Notably, transplantation of autologous LSCs has been shown to achieve significantly higher success rates and lower incidence of complications compared to allograft transplantation [7].

In LSCD, the normal immune privileged state of the cornea [8] is disrupted due to the breakdown of limbal barrier function. This disruption of the LSC niche renders LSC transplants vulnerable to immune attack. In allogeneic transplantation, the incompatible donor cells are commonly rejected by the host adaptive immune system based on the expression of highly polymorphic human leukocyte antigens (HLAs) on the cell surface [8, 9]. Therefore, the graft rejection-related problems and the limited availability of suitable donors represent major hurdles for successful allogeneic LSC transplantations. To address this problem, alternative autologous tissue sources such as hair follicle and dental pulp stem cells, oral mucosal epithelia, and mesenchymal stem cells have been investigated for their potential for corneal regeneration upon transplantation [5, 6, 10]. However, despite eliminating the need for immunosuppression, incomplete differentiation towards the corneal epithelial phenotype may pose a problem for using alternative autologous tissue grafts [10].

Differentiation of corneal cells from human pluripotent stem cells (hPSCs), namely embryonic stem cells (ESCs) and induced pluripotent stem cells (iPSCs), has emerged as a promising option for the treatment of bilateral LSCD [6, 11]. The first such protocol was described by Ahmad and colleagues in 2007 [12]. Since then, we and others have invested in developing techniques to produce an unlimited source of LSCs from hPSCs [6, 13–17]. While many of the earlier methods relied on animal-derived and poorly standardized culture components complicating their clinical translation, several clinically acceptable protocols have recently been developed [18–20]. For example, in Japan, iPSC-derived corneal transplants were recently administered to four patients suffering from bilateral LSCD [21]. However, the outcomes of these transplants are not yet known.

In addition to immunoregulation, the success of LSC therapies is highly dependent on the differentiation potential of the transplanted cells, including their ability to home to the limbus and function as native LSCs. Due to their somewhat ambiguous nature, the identification of LSCs typically relies on the simultaneous expression of several putative LSC markers combined with the absence of corneal differentiation marker CK12 [22]. For the time being, the transcription factor p63 remains the only marker with proven association with clinical success of LSC transplants [23]. Still, nuclear localization of p63 impairs its utilization for the surface marker-based LSC isolation. Numerous additional markers have been studied for their potential in identifying the therapeutically relevant *bona fide* LSCs in humans, including ABCG2 and CK15 [24–26]. In 2014, Ksander et al. [27] demonstrated that in the corneal limbus, the membrane-expressed protein ABCB5 marks a population of slow-cycling basal cells that also express ∆Np63α, the most cornea-associated isoform of p63 [28, 29]. Transplantation of ABCB5^+^ limbal cells in mice with induced LSCD lead to the long-term restoration of clear avascular corneas and absence of LSCD characteristics, while ABCB5^−^ transplants failed to do so [27]. Taken together, ABCB5 was admissibly proposed as the first prospective marker allowing selective isolation and enrichment of the therapeutically relevant LSCs. Indeed, ABCB5 has been recently utilized for enrichment of LSCs obtained both from corneal tissue of cadaveric donors [30] and iPSC-derived corneal organoids [31]. Similar to their tissue-derived counterparts, the iPSC-derived ABCB5^+^ LSCs displayed high self-renewal potential and appropriate stratification towards CK12^+^ CE in vitro [31].

In addition to self-renewal and stratification, wound healing represents one of the critical functions of healthy CE. In the case of minor wounds, the repair of surface CE is thought to be carried out without recruitment of LSCs, while excessive injuries require compensatory proliferation of LSCs and their transiently amplifying cell (TAC) progeny [2, 32]. In many epithelial tissues including CE, nucleotide-induced increase of cytosolic calcium (Ca^2+^) is known to act as the main initiator to the complex wound healing cascades [33]. In general, extracellular adenosine triphosphate (ATP), released by the cells on the injury site, binds to P2 purinergic receptors, especially P2Y2, stimulating a fast release of intracellular Ca^2+^ from their stores [33–36]. The elicited calcium wave propagates away from the wound border, triggering various downstream pathways and subsequent wound closure.

In the current study, we performed the characterization and in vitro assessment of the functional properties of hPSC-derived LSCs (hPSC-LSCs) generated using the previously established clinically applicable protocol [18, 19, 37] in comparison to tissue-derived ex vivo cultured LSCs.

## Methods

### Study design

**Fig. 1:**
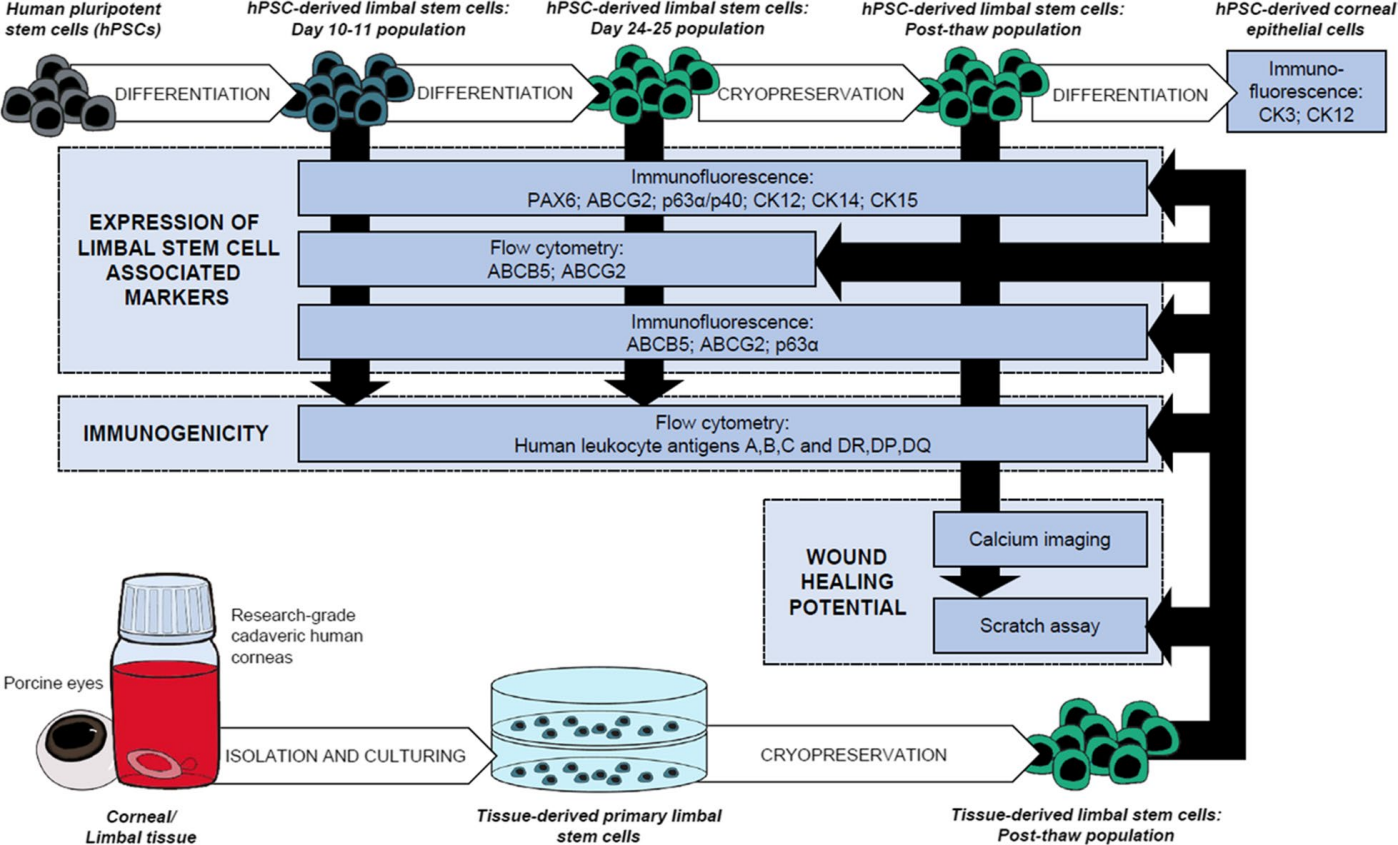
Schematic presentation of the study design. Human pluripotent stem cells (hPSCs) were differentiated towards limbal stem cells using the method described in Hongisto et al. [18], and the resulting populations were compared with tissue-derived limbal stem cells in various time points for their limbal stem cell and immunogenicity related marker expressions, using immunofluorescence and flow cytometry. Cryopreserved hPSC-derived limbal stem cells were additionally analyzed for their wound healing potential via calcium imaging and scratch assay

To compare properties of hPSC-LSCs with their tissue-derived counterparts, human and porcine LSC cultures were established in defined and feeder-free conditions and used as controls. Immunofluorescence (IF), flow cytometry (FC), scratch assay and Ca^2+^ imaging were used to study the marker expression, immunophenotype and wound healing properties of the hPSC-LSCs. Schematic overview of the study design is presented in Fig. 1.

### Isolation and culture of tissue-derived human and porcine limbal stem cells

Primary LSC cultures were established from human (h) and porcine (p) corneo-limbal tissues. Cadaveric donor human corneas were received from the Cell and Tissue Bank Regea (Tampere University) following rejection from the clinical use. Whole porcine eyes were obtained from a local slaughterhouse and transported to the laboratory on ice, where the eyes were sterilized, and corneas separated from the extra tissues as described previously [38, 39]. 2–3 mm wide limbal sections were dissected off the corneas and cut into 2 × 2 mm pieces. Limbal pieces were placed into CnT-07 medium (CELLnTEC) supplemented with 50-U/mL Penicillin–Streptomycin (pen/strep, Gibco, Thermo Fisher Scientific) and 1 mg/mL collagenase type II (Gibco, Thermo Fisher Scientific), and incubated 16–18 h at + 37 °C in the incubator. For porcine samples, limbal sections from 4 eyes were pooled, while human samples were handled individually.

Final digestion of the pieces was carried out with TrypLE Select enzyme (Gibco, Thermo Fisher Scientific) 10 min at + 37 °C. Cells released in a suspension were collected to a separate tube containing CnT-07 medium and Defined Trypsin inhibitor (DTI; Gibco, Thermo Fisher Scientific), centrifuged 5 min at 300 rcf and finally resuspended into CnT-07 medium for counting. Isolated single cells were plated onto 6-well plates or cell culture dishes coated with 5 µg/cm^2^ human placental collagen type IV (Col IV; Sigma-Aldrich) and 0.5 µg/cm^2^ human recombinant laminin-521 (LN521; Biolamina) into CnT-07 medium supplemented with 50-U/mL pen/strep. Primary cells were thereafter cultured changing the medium three times a week. Occasionally, overgrowth of fibroblastic cell types was removed by treating the cells with Tryple Select 2–3 min at + 37 °C. Upon subconfluence, primary LSCs were detached with Tryple Select and DTI, counted and cryopreserved into CnT-07 medium supplemented with 10% dimethyl sulfoxide (DMSO; Sigma-Aldrich) as a cryoprotectant. Secondary LSCs at passage 1 were recovered from cryostorage and used as controls in the experiments. “Tissue-derived LSCs” used in the text hereafter refer to these cultured secondary LSCs at p1.

### Culture of human pluripotent stem cells

Three hPSC lines, including one hiPSC and two hESC lines, previously established in our laboratory, were used for LSC differentiations. The WT001.TAU.bB2 hiPSCs were reprogrammed from healthy donor peripheral blood monocytes (PBMCs) using the CytoTune-iPS Sendai Reprogramming kit (Thermo Fisher Scientific) and characterized as previously described [40]. Derivation and characterization of the hESCs lines Regea 08/017 and Regea 11/013 was carried out as previously described in [41]. Feeder-free culture system, described in detail by Hongisto et al. [18], was utilized for maintenance of the hPSCs. In brief, the hPSCs were cultured on well plates coated with 0.55 µg/cm^2^ LN521 in Essential 8™ Flex Medium (E8, Thermo Fisher Scientific) supplemented with the 50X supplement and 50-U/mL pen/strep. Single cell passaging onto new LN521 coatings was carried out with TrypLE Select and DTI twice a week on Mondays and Thursdays, enabling a weekend-free feeding regimen. For the experiments, the quality of hPSCs were continuously monitored for attachment, growth and morphology with a Nikon Eclipse TE2000-S phase contrast microscope (Nikon Instruments Europe B.V. Amstelveen, The Netherlands), and prior to differentiations, the hPSCs were thoroughly characterized (e.g., OCT-3/4, SSEA-3, SSEA-4, TRA-1-81 and LIN28); the normal karyotype was confirmed (G-banding service by Fimlab Laboratoriot Oy Ltd., Tampere, Finland) and cells were tested for mycoplasma negativity with Venor®GeM Classic (Minerva biolabs, Berlin, Germany) (data not illustrated).

### Differentiation of human pluripotent stem cells towards limbal stem cells

Human PSCs were differentiated towards LSCs using the previously established, defined and feeder cell-free protocol [18]. Shortly, the undifferentiated hPSCs were detached and transferred into suspension culture in the presence of 5 µM Blebbistatin (Sigma-Aldrich) to form embryoid body (EB) structures overnight. Differentiation of the EBs was guided towards surface ectoderm with 10 μM transforming growth factor (TGF)-β inhibitor SB-505124 (PeproTech) and 50 ng/ml human basic fibroblast growth factor (bFGF; PeproTech) for one day, followed by 2 days with 25 ng/ml bone morphogenetic protein (BMP)-4 (PeproTech). After the induction, the EBs were plated onto Col IV/LN521 coated surfaces into defined CnT-30 corneal differentiation medium (CELLnTEC), and thereafter maintained by changing the medium three times a week. The differentiating hPSC-LSCs were subjected to analysis at days 10–11 and 24–25 (day 0 being the formation of the EBs with Blebbistatin). For some experiments, hPSC-LSCs were cryopreserved after three weeks of adherent differentiation, following the previously described cryopreservation protocol [18, 19].

### Isolation and differentiation of ABCG2^+^ hPSC-derived limbal stem cells

Differentiation dynamics of the ABCG2^+^ hPSC-LSCs were investigated with the help of fluorescence activated cell sorting (FACS). ABCG2^+^ cells were isolated from the ABCG2-enriched hPSC-LSC population at day 11 and subjected to further differentiation on Col IV/LN521 coated well plates in CnT-30 (with pen/strep). After 17 days in continued culture, the cultures were detached and spun down on object glasses to obtain cytospin samples for IF quantification of ∆Np63α. Human PSC-LSCs from one line were used in the experiment. For further details of the technical implementation of the experiment, see Supplemental methods within Additional file 1.

### Differentiation of hPSC-derived limbal stem cells further towards corneal epithelium

Cryopreserved hPSC-LSCs were thawed and plated onto Ø13mm cell-culture treated plastic coverslips (Thermanox™, Thermo Fisher Scientific) coated with Col IV/LN521, and cultured to a confluent stage in CnT-30 (with pen/strep). Coverslips with confluent cultures were thereafter transferred to a coculture with mitotically inactivated 3T3-Swiss albino feeder cells (CCL-92, ATCC, Manassas, VA), and CnT-30 medium was supplemented with 5% fetal bovine serum (FBS) and 1 mM calcium dichloride (CaCl_2_; both from Sigma-Aldrich) to promote further differentiation. Cells were cultured in the enriched differentiation conditions for 7–21 days, changing the medium every or every other day and replacing the 3T3 feeder layers every 7 days. Samples were fixed at 7, 14 and 21-day time points and analyzed for their expression of CK3 and CK12, following the basic IF protocol described in the following chapter. Human PSC-LSCs from one line were used to demonstrate the capacity of hPSC-LSCs to differentiate further towards corneal epithelial cells. Detailed description of the experiment is provided in Supplemental methods within Additional file 1.

### Immunofluorescence

Each batch of tissue-derived human and porcine LSCs was subjected to immunofluorescence characterization (IF) for their expression of PAX6, ABCG2, ABCB5, p63α, p40, CK12, CK14 and CK15 (for detailed antibody information, see Table 1). LSCs were thawed and plated into Col IV/LN521 coated well plates into CnT-07 medium (with pen/strep) 30 000–40 000 cells/cm^2^ and let to recover for 2–4 days prior fixation with 4% paraformaldehyde (PFA; Sigma-Aldrich) 20 min at room temperature (RT). The cultures were permeabilized with 0.1% Triton-X100 (Sigma-Aldrich) 10–15 min at RT and blocked with 3% bovine serum albumin (BSA; Sigma-Aldrich) 1 h at RT, following incubations with appropriate primary and fluorochrome-conjugated secondary antibody dilutions, overnight at + 4 °C and 1 h at RT, respectively. Finally, the stained cultures were mounted with Vectashield Antifade Mounting Medium with DAPI (Vector Laboratories) and round coverslips, and representative morphology and IF images were captured with Olympus IX51 fluorescence microscope. Representative IF for hPSC-LSCs was carried out once at days 11 and 25 of differentiation (using two lines) and post-thaw at p1 (using all three lines), following the similar fixation and staining protocol as described above for tissue-derived LSCs. Additionally, double-staining of ABCB5 together with p63α was carried out for one batch of differentiating hPSC-LSCs (using one line).

**Table 1:** Antibodies used in characterization of limbal stem cell marker expression

Antibody	Host	Dilution	Manufacturer	Catalog #No
PAX6	Rabbit	1:100	Sigma-Aldrich	# HPA030775
ABCG2	Mouse	1:200	Merck Millipore	# MAB4155
ABCB5	Mouse	1:200	Frank Lab*	-
p63α	Rabbit	1:200	Cell Signaling Technology	# 4892
p40 (∆Np63)	Mouse	1:100	BioCare Medical	# ACI3066
CK3	Mouse	1:100	Invitrogen	# MA1-5763
CK12	Rabbit	1:200	Abcam	# AB185627
CK14	Mouse	1:200	R&D Systems	# MAB3164
CK15	Mouse	1:200	ThermoFisher Scientific	# MS-1068-P1
Anti-rabbit IgG, Alexa Fluor 488	Donkey	1:800	Molecular Probes	# A21206
Anti-mouse IgG, Alexa Fluor 568	Donkey	1:800	Molecular Probes	# A10037
Anti-goat IgG, Alexa Fluor 647	Donkey	1:800	Abcam	# AB150131

*Frank Lab, Division of Genetics at Brigham and Women’s Hospital, Harvard Medical School Harvard University, USA

Further differentiated hPSC-LSCs cultured coverslips were similarly analyzed for their expression of CK3 and CK12, except that nuclear staining was performed by incubating Hoechst 33342 (1:1000) 5 min at RT, after the secondary antibody incubation and ProLong Gold Antifade without DAPI (both from ThermoFisher Scientific) instead of Vectashield was used to mount the samples on object glasses. The samples were imaged with Zeiss LSM 800 confocal microscope.

### Flow cytometry

Flow cytometry (FC) was used to quantify the expressions of ABCB5, ABCG2 and HLA class I/II antigens. Expressions ABCB5 and ABCG2 were measured during differentiation of the hPSC-LSCs from day 10/11 and day 24/25 populations. At least six individual samples comprising of at least two cell lines were analyzed from both populations. Due to technical issues, double staining of ABCB5 and ABCG2 was only successfully performed for one sample from two cell lines. HLA class I (A,B,C) and class II (DR,DP,DQ) proteins were both similarly analyzed from three individual differentiation batches of two cell lines and thawed p1 hPSC-LSCs from one line. One batch of tissue-derived hLSCs was used as control for both ABCB5/ABCG2 and HLA class I/II analyses.

For FC staining, cells were detached with TrypLE Select and DTI or 2 mM ethylenediamine-tetraacetic acid (EDTA; Gibco, Thermo Fisher Scientific), counted and washed with FC wash buffer containing 0.5% BSA and 1–2 mM EDTA in DPBS. 1–2 × 10^5^ cells/100 μL per sample were divided to 5 mL sample tubes, incubated with appropriate primary antibodies 20–30 min in the dark on ice, washed three times with the washing buffer and further incubated with secondary antibody, where applicable, 30 min in the dark on ice, and washed again. Information of the used antibodies is presented in Table 2. The stained cells were immediately analyzed with the BD Accuri™ C6 flow cytometer (BD Pharmingen), collecting 10 000 events of the primarily gated population of interest. The collected data were further analyzed with the BD FlowJo™ v10.6 software.

**Table 2:** Antibodies used in flow cytometry. Manufacturer recommendations in brackets

Antibody	Conjugation	Amount	Manufacturer	Catalog #No
Ms ABCB5 mAb, clone 3C2–1D12	N/A	5 μg/mL	Frank Lab*	–
Donkey anti-Mouse IgG (H + L) secondary ab	AlexaFluor 647	2.5 μg/mL	Thermo Fisher Scientific	# A31571
Ms IgG1 isotype control, clone MOPC-21	AlexaFluor 647	5 μg/mL	Thermo Fisher Scientific	# MA5-18168
Ms anti-Human ABCG2 mAb, clone 5D3	APC	1.2 (2) μg/mL	BD Biosciences	# 561451
Ms IgG2b, k isotype control, clone 27–35	APC	20 (20) μL	BD Biosciences	# 555745
Ms anti-Human HLA-A,B,C, clone G46-2.6	APC	16 (20) μL	BD Biosciences	# 562006
Ms anti-Human HLA-DR,DP,DQ, clone Tu39	FITC	18 (20) μL	BD Biosciences	# 562008

*Frank Lab, Division of Genetics at Brigham and Women’s Hospital, Harvard Medical School Harvard University, USA

In FlowJo, the negative control was used to gate the population of interest containing the cells. After excluding doublets from the analysis, the negative vs. positive gates were set with histograms (single stained samples) or quadrant tools (double-stained samples) using 0.5% marginal. Finally, the established gates were copied to each sample of the experiment. Overview of the FC gating strategy is presented within the Results section in Fig. 3b.

### Calcium imaging

Ca^2+^ signaling responses towards treatment with agonist were investigated from thawed p1 hPSC-LSCs. Cryopreserved hPSC-LSCs were thawed onto Col IV/L521 coated Ø13mm cell-culture treated plastic coverslips into CnT-30 (with pen/strep) and cultured to a confluent stage prior the analysis. Final analyses were carried out once for one line.

For imaging, samples were washed twice with pre-warmed Elliot buffer (pH 7.4 at + 37 °C, containing 137 mM NaCl, 5 mM KCl, 0.44 mM KH_2_PO_4_, 20 mM HEPES, 4.2 mM NaHCO_3_, 5 mM Glucose, 1.2 mM MgCl_2_ and 2 mM CaCl_2_, osmolarity 330). Samples were loaded with 4 μM Ca^2+^-sensitive dye fluo-4-acetoxymethyl ester (Fluo-4, ThermoFisher Scientific) in Elliot buffer together with 2.5 mM Probenecid (ThermoFisher Scientific) 30 min in + 37 °C no-CO_2_ humidified incubator. After loading, samples were washed twice with Elliot buffer and let to rest 15 min in + 37 °C no-CO_2_ humidified incubator before imaging. Ca^2+^ imaging was carried out with Nikon Eclipse FN1 upright fluorescence microscope using water dipping Apo LWD 25X objective. Samples were maintained in constant flow of fresh pre-warmed Elliot buffer throughout the imaging, temperature ranging between + 32 and + 37 °C. For detecting Ca^2+^ response towards the agonist, samples were perfused with 100 µM ATP diluted in Elliot buffer 2 min at 2:00–4:00 during the 10 min recording. Three parallel samples were imaged. Extra coverslips were fixed with 4% PFA 20 min at RT and stained for P2Y2 (Thermo Scientific, #PA1-46150, 1:200) in IF. Images of stained cells on coverslips were captured with a confocal Zeiss LSM 800 microscope.

ImageJ Image Processing and Analysis tools [42] were used to process Ca^2+^ imaging videos for ATP response analysis. Individual single cells were manually outlined as regions of interest (ROIs) (see Fig. 5c in the Results-section) and their average intensity values were extracted as a function of time using the ImageJ Multi measure tool. Raw intensity data were analyzed and normalized curve plots representing single cell responses were extracted using a custom self-developed MATLAB script package (MATLAB R2017b, The MathWorks Inc.), as reported previously [43, 44]. For quantifying cells that expressed Ca^2+^ oscillations, kymographs were extracted from 5 randomly selected segments from each parallel sample video using the command Slice [/] in ImageJ (see Fig. 5d in the Results-section). Finally, rows indicating single cell borders and intensity peaks were manually appointed. Cells expressing two or more peaks after the initial ATP-induced intensity peak, were considered to oscillate.

### Scratch assay

Scratch assays were carried out to investigate the wound healing potential of the thawed p1 hPSC-LSCs in comparison to tissue-derived pLSCs. Cryopreserved hPSC-LSCs or pLSCs were thawed onto Col IV/L521 coated wells into CnT-30 or CnT-07 medium (with pen/strep), respectively, and cultured to a confluent stage prior the analysis. Three to four wells per analysis were scratched using a 1 mL pipette tip, washed once with the culture medium, and subjected to automated time-lapse imaging at 30 min intervals for the next 24 or 48 h. The imaging was carried out using Cell-IQ® automated cell culture and analysis system or EVOS® FL Auto Cell Imaging System (ThermoFisher Scientific), using a 10 × objective. Three independent batches of hPSC-LSCs from one cell line were imaged separately for the 24-h analyses (replicate experiments (R)1–3). In R3, one batch of tissue-derived pLSCs was imaged in parallel with hPSC-LSCs. For the 48-h analysis, two independent hPSC-LSC batches were imaged in parallel with two independent tissue-derived pLSC batches (R4/5).

ImageJ Image Processing and Analysis tools [42] were used for determining the scratch closure rate from images captured at 0, 2, 6, 12, 18, 24, 30, 36, 42 and 48 h. The scratched areas at each time point were manually selected as ROIs and ROI areas (A) were calculated. The percentage of scratch closure achieved in each time point (x) was calculated using the following formula: [*A*(0 h) − *A*(*x*)]/*A*(0 h)*100.

### Statistical methods

All data are presented as the mean ± standard deviation (SD). Statistical analyses were performed with GraphPad Prism 5 software (GraphPad Software Inc.). Kolmogorov–Smirnov test was used to determine normal distribution of the data before statistical comparisons. If passed, paired T-test was utilized to compare differences between two time points in a group, or unpaired T-test was used to compare differences between two groups. When the data were not normally distributed, Mann–Whitney U test was used. Differences were considered statistically significant when *P* ≤ 0.05. Number of subjects (*n*) is constituted of hPSC-LSC differentiation or tissue-derived LSC isolation batches, each of individual cell batches being considered as biological replicates.

## Results

### Expression of limbal stem cell-associated markers in differentiating hPSC cultures in comparison to tissue-derived limbal stem cells

**Fig. 2:**
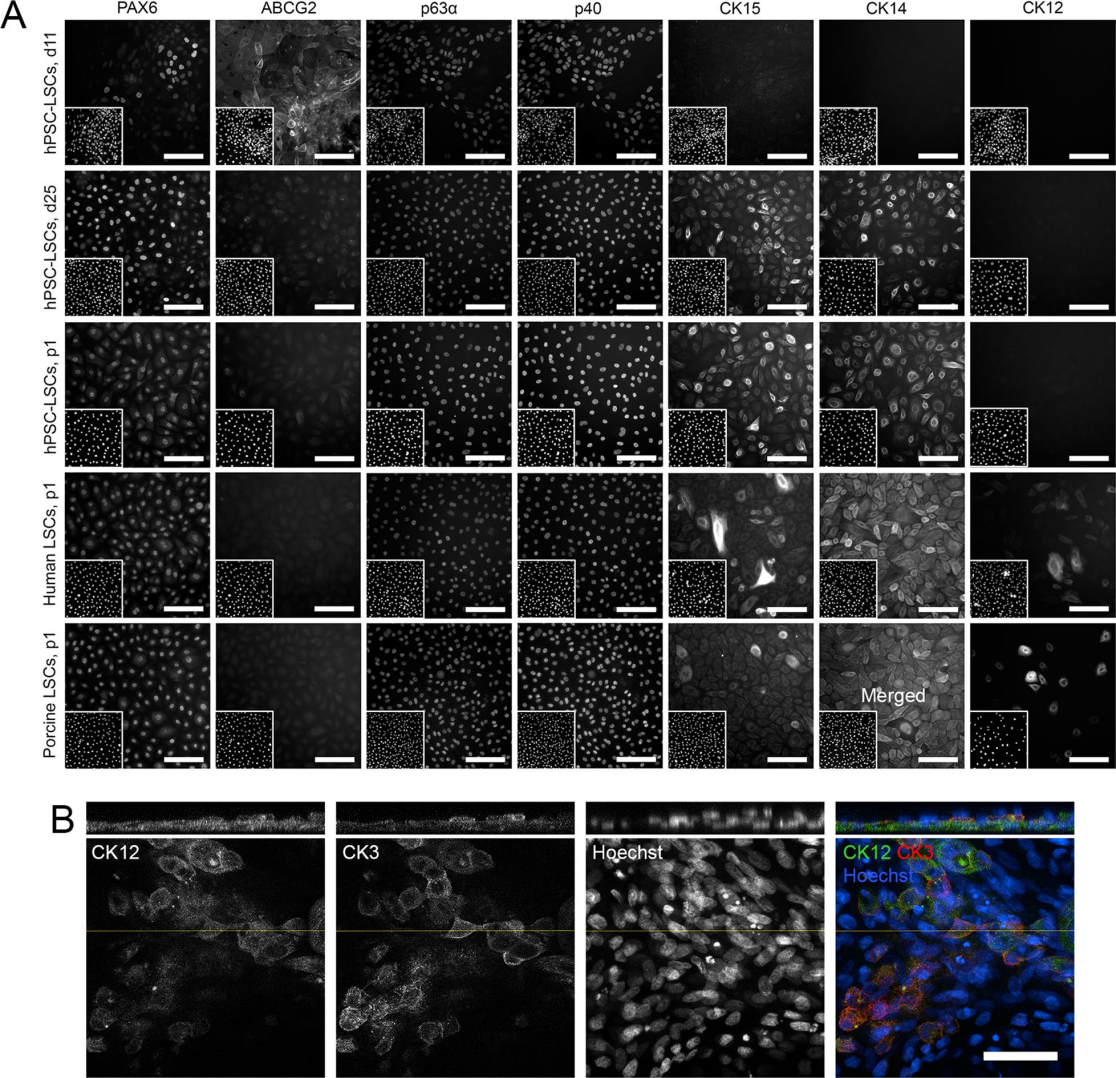
Expression of limbal stem cell associated markers. **a** Representative immunofluorescence images of hPSC-derived LSCs at day (d)11, d25 and at passage 1 (p1) post-thaw, in comparison to human and porcine LSCs at p1 post-thaw. Corresponding DAPI-stained nuclei presented in the lower left corner of each panel. Scale bars, 100 μm. Representative hPSC-LSC data shown for line Regea08/017. **b** Cytokeratin (CK)12 and CK3 expression and cell layering in p1 hPSC-LSCs after 21 days further differentiation in CnT-30 supplemented with 5% FBS and 1 mM CaCl_2_. Scale bar, 50 μm. CK12/CK3 expression data for hPSC-LSC line Regea08/017

Immunofluorescence analysis of several LSC-associated markers demonstrated the increased expression of PAX6, ∆Np63α detected by p63α/p40 co-staining, CK14 and CK15 during hPSC differentiation at day 11 and 25. In these cultures, ABCG2 was only detected at day 11, and the corneal epithelial differentiation marker CK12 was not present at day 11 or day 25 (Fig. 2a). A similar marker expression was observed in cryopreserved p1 hPSC-LSCs (Fig. 2a). However, arising expression of CK3 and CK12 was observed after further epithelial differentiation of post-thaw p1 hPSC-LSCs in enriched differentiation conditions in CnT-30 supplemented with FBS and CaCl_2_ (Fig. 2b, Additional file 2: Fig. S1), along with cell layering (Fig. 2b). The tissue-derived cultured hLSCs and pLSCs displayed robust expression of PAX6, ∆Np63α, CK14 and CK15, low levels of CK12 and no detectable ABCG2 (Fig. 2a). These staining patterns resembled day 25 and p1 hPSC-LSC cultures (Fig. 2a). Within hPSC-LSCs, both hESC- and hiPSC-derived LSCs demonstrated comparable marker expression patterns throughout the differentiation process and post-thaw recovery (data not illustrated).

### ABCB5 marks the population of ABCG2-negative and p63α-positive hPSC-LSCs

**Fig. 3:**
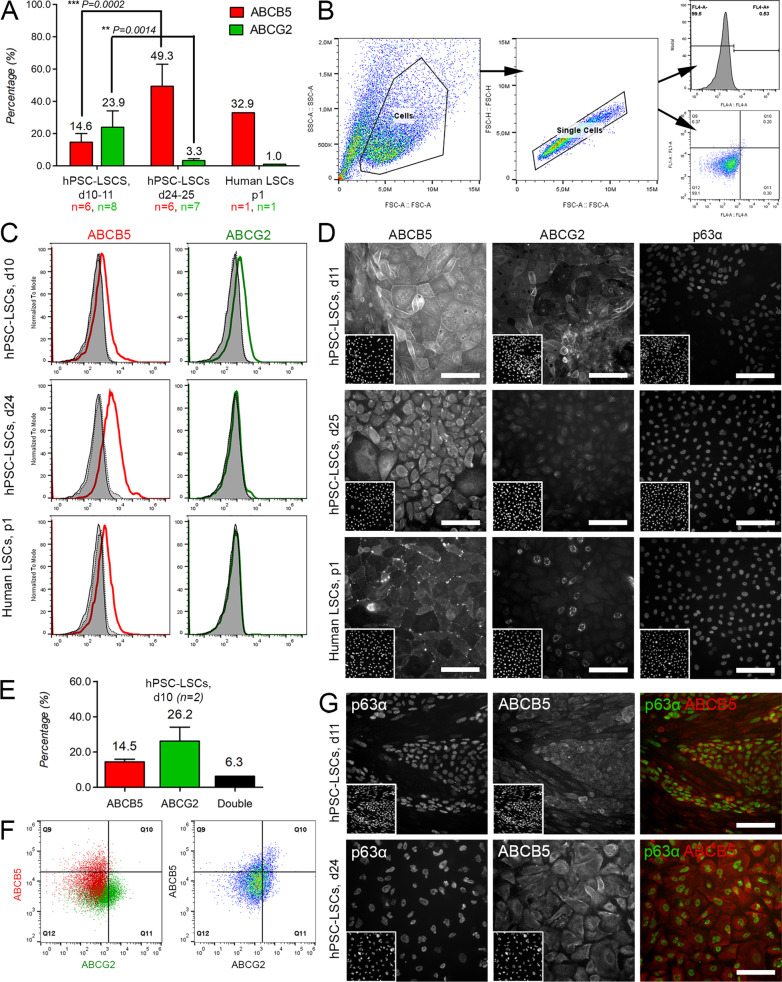
Expression of ABCB5, ABCG2 and p63α. **a** Flow cytometry (FC) analyses of ABCB5 and ABCG2 expression in day (d) 10–11 (*n*_ABCB5_ = 6, n_ABCG2_ = 8) and d24-25 (n_ABCB5_6, n_ABCG2_ = 7) hPSC-LSCs populations and in human LSCs at passage (p)1 (*n* = 1). For statistical analyses, paired t test and Mann–Whitney U test were used for comparison of day 10–11 and day 24–25 hPSC-LSC populations. **b** Gating strategy used in FC. **c** Representative FC histograms showing unstained (tinted black) and isotype-stained controls (dotted black) in overlays with ABCB5 (red) and ABCG2 (green) stained populations. **d** Immunofluorescence (IF) analysis of single-stain ABCB5, ABCG2 and p63α. Corresponding DAPI-stained nuclei presented in the lower left corner of each panel. Scale bars, 100 μm (applies for d and g). **e** FC results of ABCB5/ABCG2 double-stained cells in d10 hPSC-LSCs. **f** Representative FC dot plots of the single- and double-stained d10 hPSC-LSCs. **g** IF analysis of ABCB5/p63α double staining in d11 and d25 hPSC-LSCs. Representative hPSC-LSC data in all images shown for line Regea08/017

Flow cytometry analyses revealed divergent expression patterns of ABCB5 and ABCG2 during differentiation of hPSC-LSCs (Fig. 3a). Gating strategy and representative flow cytometry histograms for both markers, accompanied with negative and isotype controls, are shown in Fig. 3b, c. In the course of differentiation, expression of ABCB5 significantly increased from 14.7 ± 5.3% at day 10–11 to 49.3 ± 13.7% at day 24–25, (*n* = 6, *P* = 0.0002, unpaired t-test) while expression of ABCG2 significantly decreased from 23.9 ± 10.2% at day 10–11 (*n* = 8) to 3.3 ± 1.8% by day 24–25 (*n* = 7, *P* = 0.0014, Mann–Whitney U test) (Fig. 3a). In cultured tissue-derived hLSCs, ABCB5 was expressed by 32.9% and ABCG2 by 1.0% of the cells (*n* = 1) (Fig. 3a). Immunofluorescence analyses revealed distinct ABCB5 expression patterns in day 10–11 hPSC-LSCs, day 24–25 hPSC-LSCs and tissue-derived hLSCs (Fig. 3d). ABCB5 staining in p1 post-thaw hPSC-LSCs was similar to day 24–25 hPSC-LSCs (data not illustrated). ABCG2 was detected in day 10–11 hPSC-LSC cultures with significantly reduced expression in day 24–25 hPSC-LSCs or tissue-derived hLSCs (Fig. 3d). High ∆Np63α expression determined by p63α and p40 positivity was observed in the majority of day 24–25 hPSC-LSCs and hLSCs, while a smaller number of p63α/p40 double-positive cells was observed in day 10–11 hPSC-LSC cultures (Fig. 3d, staining shown for p63α). Importantly, differentiation of isolated ABCG2^+^ cells yielded a hPSC-LSC population with high expression of ∆Np63α (88.7 ± 2.8%, *n* = 1565 cells, see Additional File 2: Figure S2).

Double staining flow cytometry analyses revealed ABCB5 expression by 14.5 ± 1.5% and ABCG2 expression by 26.2 ± 7.9% of cells in day 10 hPSC-LSCs cultures (Fig. 3e, f), among which 6.3 ± 0.04% of cells co-expressed both proteins (*n* = 2). Immunofluorescence analyses demonstrated co-expression of ABCB5 and p63α in both day 11 and day 25 hPSC-LSCs (Fig. 3g). This was especially highlighted in the day 11 hPSC-LSC culture, where a clear separation to ABCB5/p63α double-positive and double-negative populations was observed, while in day 25 hPSC-LSCs the expression of both markers was more ubiquitous (Fig. 3g).

### Immunophenotype of hPSC-LSCs

**Fig. 4:**
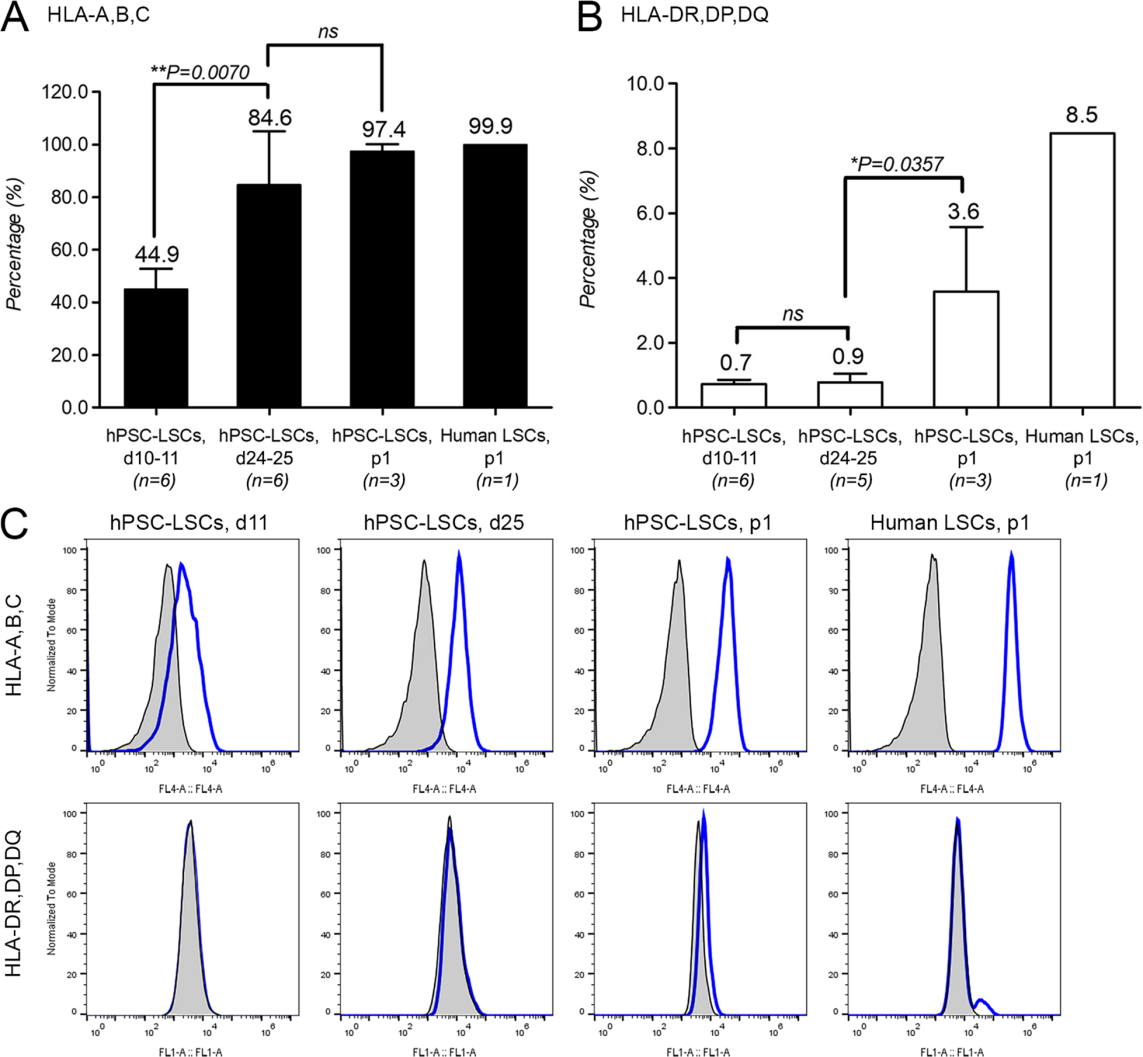
Expression of human leukocyte antigen (HLA) class I and class II proteins. **a** Flow cytometry (FC) results of HLA-A,B,C (class I) and **b** HLA-DP,DR,DQ (class II) expression in day (d) 10–11 (*n* = 6), d24–25 (n_HLA-A,B,C_ = 6, n_HLA-DR,DP,DQ_ = 5) and passage 1 (p1, *n* = 3) hPSC-LSCs populations, and in p1 human LSCs (*n* = 1). For statistical analysis, paired t-test was used for comparison of day 10–11 and day 24–25 hPSC-LSC populations, and Mann–Whitney U-test was used for comparison of day 24–25 and p1 hPSC-LSC populations. **c** Representative FC histograms showing unstained (tinted black) controls in overlays with HLA-A,B,C- or HLA-DR,DP,DQ-stained populations. Representative hPSC-LSC data shown for line Regea08/017

Flow cytometry was used to analyze the expression of HLA class I (A,B,C) (Fig. 4a) and class II (DR,DP,DQ) (Fig. 4b) proteins during differentiation of hPSC-LSCs. At day 10–11, class I HLAs were expressed on 44.9 ± 7.9% of the cells and their expression was significantly increased to 84.6 ± 20.5% on day 24–25 (*n* = 6, *P* = 0.0070, paired T-test) (Fig. 4a). Noteworthy, especially in one replica experiment where the two parallel cell lines gave highly varied results (90.9% in Regea08/017 hESC-LSCs vs. 53.4% in WT001.TAU.bB2 hiPSC-LSCs) at day 24–25, lower differentiation efficacy was also clearly observed during morphological evaluation correlating with the lower expression of HLA I (data not illustrated). Further, class I HLAs were expressed rather uniformly in 97.4 ± 2.8% of p1 hPSC-LSCs post-thaw (*n* = 3), but difference to day 24–25 hPSC-LSCs was not significant (Mann–Whitney U test). Similar uniform expression of HLA class I (99.9%, *n* = 1) was measured in tissue-derived, cultured hLSCs, but as seen in representative FC histograms in Fig. 4c, the mean fluorescence intensity (MFI) shown on x-axis is markedly lower in hPSC-LSCs in comparison to tissue-derived hLSCs.

On the other hand, HLA class II proteins were not expressed by day 10–11 hPSC-LSC cultures (0.7 ± 0.1%) and their expression remained at negligible levels at day 24–25 (0.9 ± 0.3%) (Fig. 4b). Interestingly, post-thaw p1 samples showed slightly but significantly higher expression of HLA class II (3.6 ± 2.0%) in comparison to day 24–25 samples (*n* ≥ 3, *P* = 0.0357, Mann–Whitney U test). In hLSCs, class II HLAs were expressed in 8.5% of the cells. Representative FC histograms of the studied populations are shown in Fig. 4c.

### Wound healing properties of hPSC-LSCs

**Fig. 5:**
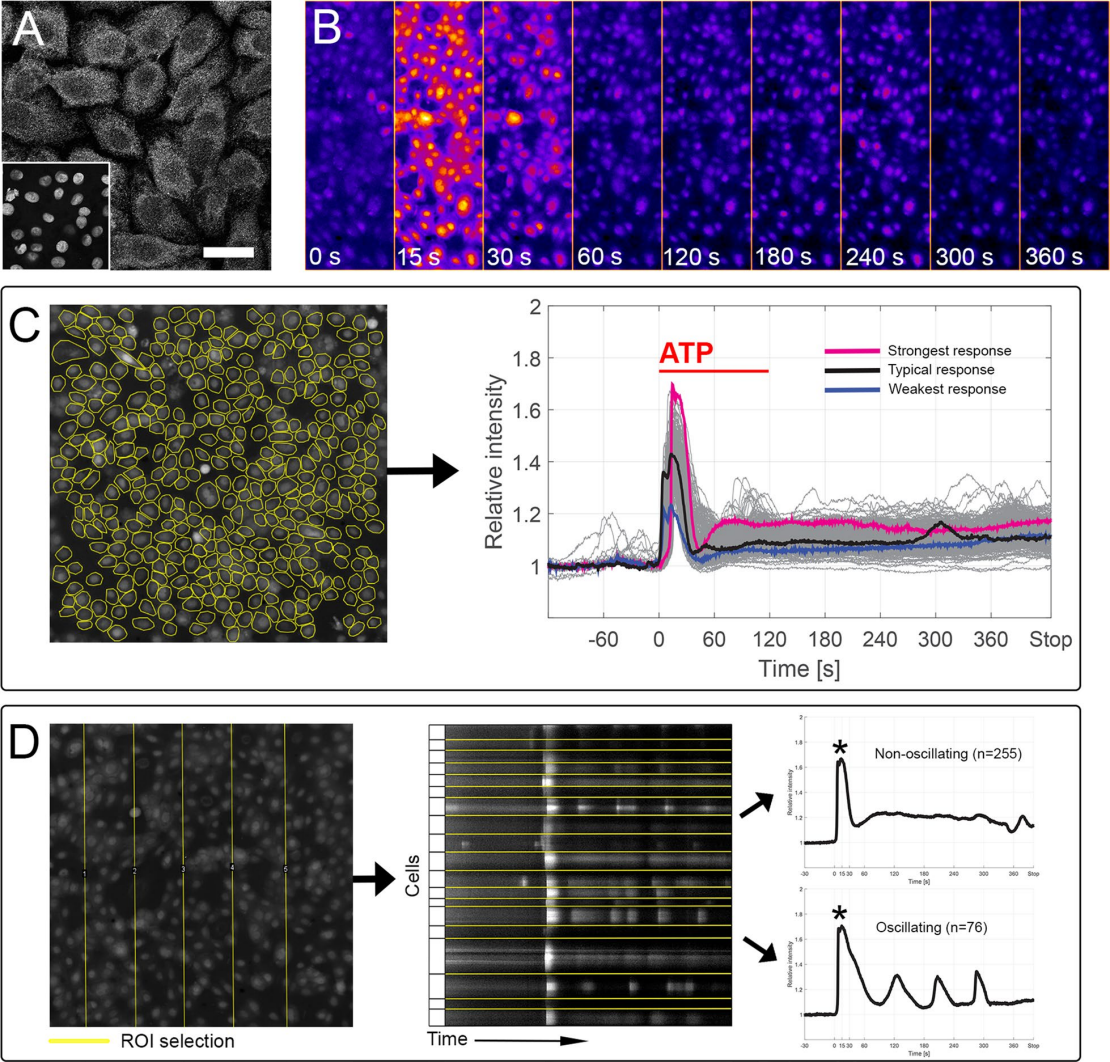
Calcium signaling properties. **a** Expression of purinergic receptor P2Y2 in p1 hPSC-LSCs. Scale bar, 20 μm. **b** Representative pseudocolored image time series of ATP-induced Ca^2+^ response in p1 hPSC-LSCs, visualized with Fluo-4 Ca^2+^ indicator. **c** Representative single cell selections from one parallel sample and corresponding curve cluster showing relative intensities as a function of time for each analyzed cell in a sample. Typical response (based on relative intensity peak during the ATP stimulation) is highlighted in black, strongest response in pink and weakest response in blue.** d** Representative regions of interest (ROIs) from one parallel sample and resulting kymograph from one representative ROI, with example curves of single non-oscillating and oscillating p1 hPSC-LS cells. Asterix marks the primary ATP-induced calcium peak. All data for hPSC-LSC line Regea08/017

The functional potential of the post-thaw p1 hPSC-LSCs was evaluated through examination of calcium signaling and wound healing capacity. Immunofluorescence analyses revealed abundant expression of the purinergic receptor P2Y2, the main mediator of ATP-induced Ca^2+^ response (Fig. 5a). Accordingly, robust Ca^2+^ response was observed after agonizing hPSC-LSCs with the P2Y2 ligand ATP, as visualized in a pseudocolored image time series in Fig. 5b. Of 1082 analyzed cells, 99.7% were responsive to ATP stimulation. The cells produced responses with both fast rise and fast decay (Fig. 5c). In addition to the main Ca^2+^ peak, many of the individual cells displayed post-stimulatory Ca^2+^ oscillations. In total, 331 cells were analyzed for their oscillatory behavior and 23.8 ± 15.9% of those produced two or more separate Ca^2+^ peaks after the initial response (Fig. 5d).

**Fig. 6:**
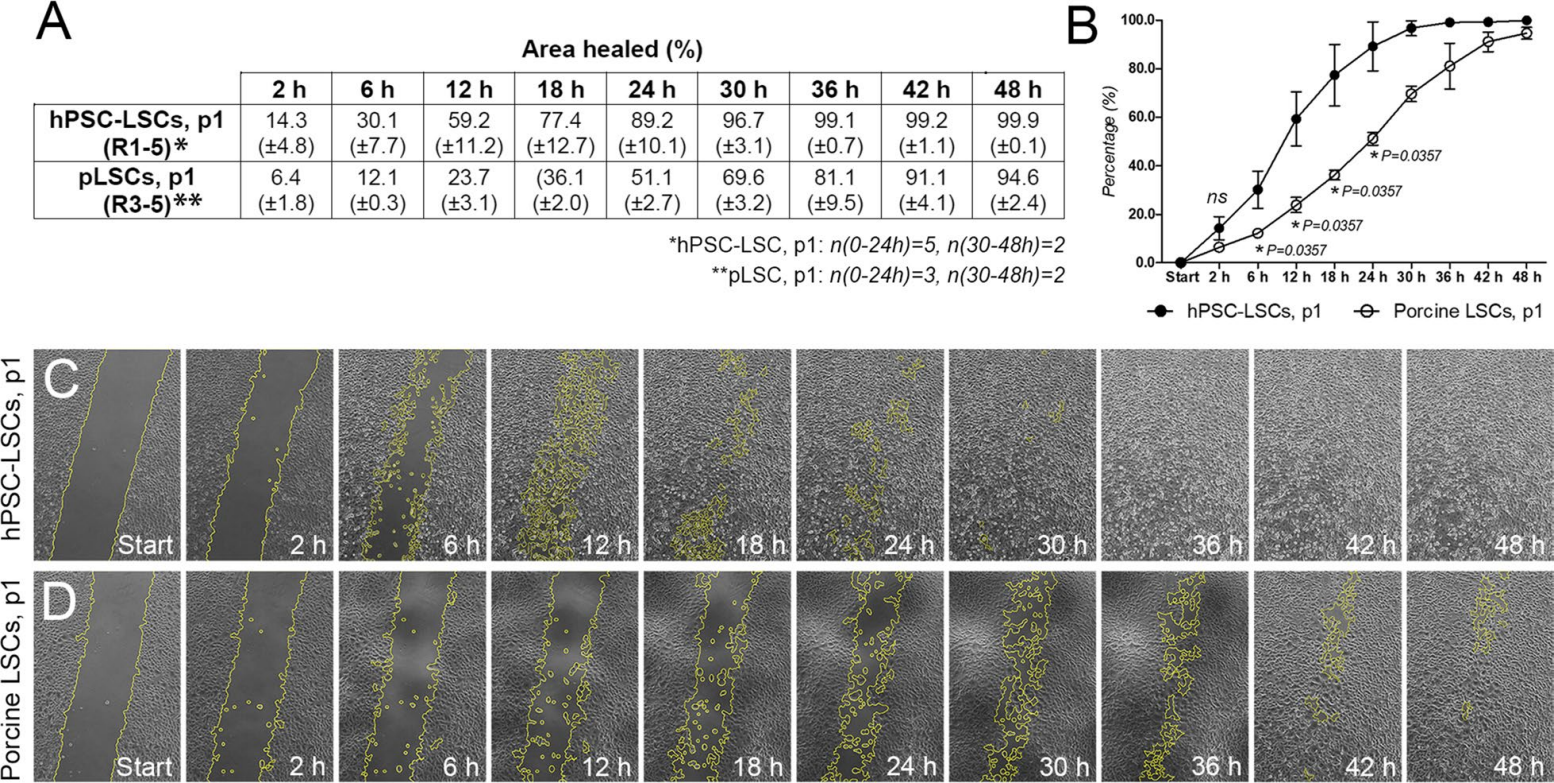
Wound healing capacity. **a**, **b** Scratch assay results for passage 1 (p1) hPSC-LSCs and p1 porcine LSCs. Results are presented as mean (± standard deviation) of all individual replicate experiments. For statistical analysis, Mann–Whitney U-test was used to compare between the populations in 2–24 h time points. **c** Representative phase contrast images of p1 hPSC-LSC and **d**, p1 porcine LSC scratches at 0, 6, 12, 18, 24, 30, 36, 42 and 48 h time points in R4/5. Yellow lines mark the borders of analyzed wound area. All hPSC-LSC data for line Regea08/017

The wound healing capacity of p1 hPSC-LSCs was evaluated in the scratch assay [45]. Wound closure rate for post-thaw p1 hPSC-LSCs (n_2-24 h_ = 5 and n_30-48 h_ = 2) was on average 14.3 ± 4.8% at 2 h, 30.1 ± 7.7% at 6 h, 59.2 ± 11.2% at 12 h, 77.4 ± 12.7% at 18 h, 89.2 ± 10.1% at 24 h and > 95% at 30 h and later time points (Fig. 6a, b). For tissue-derived, cultured pLSCs (n_2-24 h_ = 3 and n_30-48 h_ = 2) the corresponding wound closure rates were 6.4 ± 1.8%, 12.1 ± 0.3%, 23.7 ± 3.1%, 36.1 ± 2.0%, 51.1 ± 2.7%, 69.6 ± 3.2%, 81.1 ± 9.5%, 91.1 ± 4.1 and 94.6 ± 2.4%, respectively (Fig. 6a, b). The healed area of hPSC-LSCs was on average 55.2%, 59.2%, 60.0%, 53.4%, 42.7%, 28.0%, 18.2%, 8.2% and 5.3% larger than that of pLSCs by 2, 6, 12, 18, 24, 30, 36, 42 and 48 h. The differences were significant (*P* = 0.0357, Mann Whitney U test) between 6 and 24 h (Fig. 6b). Human PSC-LSCs also displayed more migratory behavior in comparison to pLSCs (Fig. 6c, d, for more comprehensive demonstration see also the Supplementary movie S3 provided in Additional File 2).

## Discussion

The development of safe, efficient, and economically feasible tissue-engineered grafts to treat LSCD is an important objective in the battle against insufficiency of the currently available methods, especially in case of bilateral LSCD. Here, we have investigated the marker expression of LSCs produced from hPSCs with the previously established defined and feeder-free method [18, 19] and provide insight of their functional potential in wound healing.

The LSC associated marker expression profile and its maintenance after cryopreservation in hPSC-LSCs were in line with our previous observations, including the expression pattern of ABCG2 with bright membrane-localized staining at day 11 and negligible expression in the following time points [18, 37]. The results demonstrate high reproducibility of the method in our hands. Noteworthy, Sun et al. [46] utilized a differentiation protocol with the same induction molecules to investigate cellular heterogeneity during differentiation of hPSC-LSCs at days 0, 7, 14 and 21, using single cell RNA sequencing. Interestingly, they reported overall very low expressions for PAX6, p63, and CK14 and distinctive expression pattern for ABCG2, demonstrating the highest peak of ABCG2 expression at day 21. These differences could be explained by divergent culture methods affecting cell signaling pathways, such as the use of Matrigel in hPSC culture and the use of ROCK inhibitor instead of blebbistatin in EB formation. However, the results highlight the importance of the ABCG2^+^ cell population in the hPSC-LSC cultures.

In the current study, the expression of another LSC marker ABCB5 during hPSC-LSC differentiation was also investigated. Both ABCB5^+^ [31] and ABCG2^+^ [24, 25] cells have been shown to possess the capacity to form holoclones, which identifies the self-renewing stem cells in vitro. Furthermore, we have previously associated high expression of ABCG2 with the presence of a quiescent subpopulation, as well as with increased regenerative potential of hPSC-LSCs [37]. In the current study, we found that during hPSC-LSC differentiation, expression of ABCB5 was significantly increased from day 10–11 (14.6%) to day 24–25 (49.3%). Previously, using the exact protocol, we found that ∆Np63α expression also increased from 23.2% to 54.3% at these time points [37]. Due to their critical role in LSC functionality, and preferred coexpression of p63α and ABCB5 in vivo [27], quantification of PAX6/p63 double-positive cells was used as potency assay during quality control of the novel ABCB5^+^ LSC-based ATMP [30]. Importantly, double staining of ABCB5 and p63α in our study showed that these markers were coexpressed both on day 11 and day 25 hPSC-LSCs cultures. It is noteworthy that, while expression of the ∆Np63α isoform of the most widely used LSC marker p63 (first proposed by Pellegrini et al. in 2001 [47]) can be shown only via double staining with p63α and p40 antibodies, we have previously demonstrated that 90% of the hPSC-LSCs expressing either p63α or ∆Np63 are double-positive with the other marker as well [18]. Thus, in the context of this paper it is reasonable to assume that positivity to either of those markers represents the presence of specific ∆Np63α isoform.

In contrast to ∆Np63α and ABCB5, expression of ABCG2 in hPSC-LSCs declined during differentiation to 3.3% of cells by day 24–25. At day 10–11, both ABCB5 and ABCG2 were expressed, by 14.5% and 26.2% of the cells, respectively, and a small subset of 6.3% of all cells coexpressed both proteins. Interestingly, a population of highly regenerative ABCB5^+^/ABCG2^+^ LSCs could also be retrieved from human limbus using a specialized culturing process [48], raising an interest towards the therapeutic relevance of this earlier population as well. In a transcriptomic analysis investigating the stemness hierarchy of human LSCs expressing ABCB5 and p63, ABCB5 was positioned after p63 [49]. Here, ABCG2^+^ cells isolated from day 11 hPSC-LSC cultures gave rise to populations with high (> 88%) expression of ∆Np63α. This is in line with our previous results, where similarly sorted ABCG2^+^ cells produced > 99% pure ABCB2^−^/p63α^+^ population [37]. While we have not examined whether p63 is co-expressed in some of the isolated ABCG2^+^ cells at day 10–11, these results and the transient expression of ABCG2 during the early phase of hPSC-LSC differentiation suggests its role as a preceding marker in relation to p63 and potentially ABCB5, both of which appeared more prevalent at day 25. As known from Rama et al. 2010 [50], long-term restoration of the corneal surface is accomplished with p63^+^ LSCs, and preclinical evidence of the high potential of ABCB5^+^ LSCs [27, 30, 31] is compelling as well. Although the developmental trajectory of ABCG2, ABCB5 and ∆Np63α remain to be further studied, they all represent extremely attractive hPSC-LSC populations for therapeutic applications. Importantly, the cell surface localization of ABCG2 and ABCB5 allows antibody-dependent isolation of living cells, which can be used to purify and enrich the target population, as recently demonstrated by Norrick et al. (2021) for ABCB5 [30].

Additionally, we addressed the development of immunogenic properties during differentiation to gain preliminary insight to the future immunoregulatory aspects of hPSC-LSC transplantation. HLA class I proteins are expressed by most nucleated cells and they are recognized by cytotoxic T cells, leading to direct extermination of mismatched tissue transplants [9, 51]. HLA class II proteins are mainly expressed by antigen presenting cells (APCs), which are represented by the Langerhans cells in the cornea [8]. The Langerhans cells activate B and T cells indirectly via stimulation of helper T cells. In our investigations, the baseline expression of HLA class II antigens in hPSC-LSCS was low. The number of cells expressing class I HLAs significantly increased during differentiation, and lower/slower differentiation efficacy of one analyzed cell batch was likewise reflected to the lower expression of class I HLAs in that particular sample at day 24–25. Based on our studies, both hESC- and iPSC-LSCs express HLAs in similar manner and the differences in HLA expression levels and differentiation efficacy towards LSCs in general is more dependent on characteristics of individual cell lines rather than the source of hPSCs. Our results are in line with previously published reports stating that while hESCs and hiPSCs have low expression of class I HLAs, their expression is increased upon differentiation, even though their levels remain low in comparison to somatic cells [51, 52]. Importantly, expression of HLA class I antigens in hPSC ocular derivatives is strongly elicited when the cells are exposed to inflammatory signals, as previously reported for ESC-derived corneal epithelial cells (CECs) [53] and iPSC-derived retinal pigment epithelium (RPE) [52, 54]. However, in ESC-CECs the HLA class I staining MFI remained at significantly lower level in comparison to primary LSCs and expression of class I antigens was not affected, indicative of the lower immunogenicity of ESC-CECs [53]. Similarly to Wang et al. (2016), we also found the baseline expression of HLA class I was markedly lower in hPSC-LSCs compared to tissue-derived hLSCs. While this basic investigation of the immunogenic status of our hPSC-LSCs implicates low immunogenicity in comparison to donor tissue, a more comprehensive study including the inflammatory environment needs be carried out utilizing this specific differentiation method. Further, modern immunomodulatory strategies like utilization of “universal donor lines” with HLA inactivation [55–57] should be explored in order to minimize the immunogenic threats and promote the usability of the method.

The marker expression of differentiating hPSC-LSCs at day 10–11 and 24–25 as well as cryopreserved hPSC-LSCs at p1 were also compared with cryopreserved p1 human and porcine tissue-derived LSCs. While human corneas are only randomly available for research purposes for our group, porcine corneas serve as a validated substitute for human tissue [38, 39]. In this study, the tissue-derived cultured LSCs, both human and porcine, showed striking similarity to day 25 and p1 post-thaw hPSC-LSCs, except for CK12 expression. The presence of residual CK12^+^ mature corneal cells in tissue-derived cultures is to be expected, however their low numbers in addition to wide expression of ∆Np63α, CK15, CK14, and ABCB5 indicate, that the used feeder-free and defined culture conditions of tissue-derived LSC promote maintenance of progenitors over maturation of the cells. In hPSC-LSCs, further differentiation could be induced by culturing the cells in enriched differentiation condition including 3T3 feeders. While the formation of properly stratified multilayered CE was not achieved within the maximum 21-day observation period, preliminary cell layering and arising expression of the CE specific terminal differentiation markers CK3 and CK12 demonstrates the capacity of hPSC-LSCs to carry out corneal lineage differentiation. However, the unequivocal functional capacity of hPSC-LSCs in terms of multilayered CE regeneration remains to be verified in vivo.

In this study, the functional properties were studied in regard to wound healing, which is a fundamental function of limbal cells. Corneal wound closure is achieved by migration, proliferation, and differentiation of TACs [2, 58]. Post-thaw p1 hPSC-LSCs were chosen for the analysis because of their phenotypic similarity to the tissue-derived cultured LSCs, and because the maintenance of the functional LSC characteristics after cryopreservation is an important factor for the development of future cell therapeutics. Importantly, we were able to demonstrate that post-thaw p1 hPSC-LSCs possess the functional capability to initiate and carry out wound healing. Injury-driven release of extracellular ATP and its subsequent binding to P2Y2 receptors of nearby cells is known to trigger a propagating calcium wave and wound healing response in CE [33–36]. We confirmed the presence of P2Y2 by IF and used direct administration of P2Y2 agonist ATP to elicit Ca^2+^ response in unwounded confluent cultures of p1 hPSC-LSC. Indeed, signal detection with Ca^2+^-sensitive dye Fluo-4 demonstrated a uniform Ca^2+^ response throughout the hPSC-LSC monolayer. Rapid release and rapid restoration of Ca^2+^ from the intracellular stores allows fast reaction to repeated stimulus. While the utilized set-up does not allow to examine the dynamic propagation of the Ca^2+^ wave between the cells, the cells treated with an agonist all react in a similar manner as cells located directly next to a wound [59]. In experiments of Lee et al. 2019, the cultured corneal limbal cells located to this so-called leading edge of the wound were also shown to exhibit sustained Ca^2+^ oscillations, which we also observed in our samples. Their results suggest that sustained Ca^2+^ oscillations were required for wound closure via changes in cell shape and mobilization. The same authors also confirmed similar response in vivo in organ cultured mouse corneas, where oscillatory events were preferably localized in basal corneal epithelial cells. In another study, cultured bovine corneal endothelial cells, the Ca^2+^ oscillations were linked with potential role in modulation of the apoptotic response after mechanical injury [60]. Overall, our current Ca^2+^ imaging results strongly support the notion that post-thaw p1 hPSC-LSCs possess functional capacity to detect injury-driven signals and elicit appropriate response. Further proof of the cryopreserved hPSC-LSCs’ ability to accomplish wound healing was obtained via scratch assay. Noteworthy, the wound closure rate of hPSC-LSCs appeared to be even faster in comparison to cultured tissue-derived pLSCs. One explanation to this is the more immature status of p1 hPSC-LSCs after relatively short differentiation period, whereas tissue-derived LSCs are likely to comprise further differentiated progenitors and TACs with limited proliferative capacity. As an important consideration, even the golden standard methods for ex vivo cultivation (using amniotic membrane or 3T3 feeder layers) were recently deemed insufficient in maintaining the in vivo-associated transcriptomic profile of LSCs [61]. However, it is of interest to see if differentiation of LSCs from hPSCs could be utilized in capturing this elusive in vivo phenotype as well.

## Conclusion

In this study, we carried out further investigation of the hPSC-LSCs produced with our previously established defined and xeno-free differentiation method. Here, we provide further insight into the properties of different hPSC-LSC subpopulations and demonstrate functional potential of cryopreserved hPSC-LSCs. A more detailed analysis of the differentiation trajectory of ABCG2, ABCB5 and ∆Np63α and the potential roles of their subpopulations in LSC quiescence, self-renewal, and differentiation remain to be performed. Furthermore, it is yet to be determined which one of the emerging subpopulations is the most potent therapeutically; the early arising day 10–11 hPSC-LSC populations comprising higher number of ABCG2^+^ cells alongside some ABCB5 and p63α expression, or the late arising day 24–25 population with minimal ABCG2^+^ but abundant ABCB5^+^ and p63α^+^ cells. Specifying the hPSC-LSCs population with the highest regenerative capacity in vivo remains as an important future task, for which the LSC surface markers ABCG2 and ABCB5 as isolation tools offer great opportunities.

## Limitations of the study

In this study, we characterized the differentiation of hPSC-derived LSCs and carried out evaluation of their wound healing-associated functional potential in vitro*.* While our results provide indications of the regenerative potential of these cells, the relevance of the findings presented herein remain to be verified in vivo.

## Supplementary Information

**Additional file 1**: Containing detailed descriptions of the fluorescence activated cell sorting of ABCG2+ hPSC-LSCs and further differentiation of p1 hPSC-LSCs

**Additional file 2**: Containing supplementary data about CK3 and CK12 expression in post-thaw p1 hPSC-LSCs (Supplementary Fig. S1), ∆Np63α expression in hPSC-LSCs derived from the ABCG2+ cell population (Supplementary Fig. S2) and the Supplementary Movie S3 showing the wound closure of p1 hPSC-derived LSCs versus the tissue-derived porcine LSCs

## Data Availability

All the data used to support the findings of this study are included within the article and its additional files.

## Abbreviations

ATMP: Advanced therapy medicinal product
ATP: Adenosine triphosphate
Ca^2^^+^: Calcium
CaCl_2_: Calcium dichloride
CE: Corneal epithelium
CLET: Ex vivo cultivated limbal stem cell transplantation
ESC: Embryonic stem cell
Col IV: Collagen type IV
E8: Essential E8 Flex medium
EB: Embryoid body
FC: Flow cytometry
DMSO: Dimethyl sulfoxide
DTI: Defined Trypsin Inhibitor
ESC: Embryonic stem cell
HLA: Human leukocyte antigen
hPSC: Human pluripotent stem cell
IF: Immunofluorescence
iPSC: Induced pluripotent stem cell
LN521: Laminin-521
LSC: Limbal stem cell
LSCD: Limbal stem cell deficiency
p1: Passage 1
Pen/Strep: Penicillin/Streptomycin
PFA: Paraformaldehyde
ROI: Region of interest
RT: Room temperature
SD: Standard deviation
SLET: Simple limbal epithelial transplantation
TAC: Transient amplifying cell
