# Supplementary material for: Corneal epithelial differentiation of human pluripotent stem cells generates ABCB5+ and ∆Np63α+ cells with limbal cell characteristics and high wound healing capacity
Source: Stem Cell Res Ther. 2021 Dec 20;12:609. doi: 10.1186/s13287-021-02673-3 (PMC8691049; doi:10.1186/s13287-021-02673-3)
Supplement: Supplementary file 1 — Additional file 1: Containing detailed descriptions of the fluorescence activated cell sorting of ABCG2+ hPSC-LSCs and further differentiation of p1 hPSC-LSCs [file 13287_2021_2673_MOESM1_ESM.docx]

**SUPPLEMENTAL METHODS**

**Isolation and differentiation of ABCG2^+^ hPSC-derived limbal stem cells**

Differentiation dynamics of the ABCG2^+^ hPSC-LSCs was investigated with the help of fluorescence activated cell sorting (FACS). Differentiating Regea08/017 hPSC-hPSCs were detached with TrypLE Select and DTI, counted and washed with FC wash buffer containing 0.5% BSA and 2 mM EDTA in DPBS. Sorting samples containing up to 1 x 10^6^ cells/100µL in a 5 mL sample tube were prepared and incubated with 3 µL APC-conjugated Ms anti-Human ABCG2 mAb, clone 5D3 (#561451, BD Biosciences) 20 min on ice, protected from light. Appropriate negative and isotype control samples were prepared alongside the sorting sample.

The controls and sorting samples were analyzed with FACSAria™ Fusion cell sorter operating with the FACSDiva™ software (both from BD Biosciences, San Jose, California, USA). After identifying and gating the correct population of interest, ABCG2^+^ cells from the sorting sample were sorted directly to the 5 µg/cm^2^ Col IV/0.5 µg/cm^2^ LN521 coated 6-well plates, 10 000 cells per well in CnT-30 medium supplemented with 50-U/mL pen/strep and 10 µM Rho kinase inhibitor Y-27632 (Tocris Bioscience) for the first days after sorting. Thereafter, the cells were cultured in CnT-30 without Y-27632, changing the medium three times a week.

After 17 days the cells were detached with TrypLE Select and DTI, washed with ice-cold DPBS and ca. 100 000-150 000 cells were spun down onto an object glass, using a CellSpin II cytocentrifuge (Tharmac, GmbH, Waldsolms, Germany). The sample was immediately fixed with 4% PFA, 15 min at RT and subjected to double-IF-staining with p63α and p40 antibodies. Five representative images were captured with the Olympus IX51 fluorescence microscope and analysis of 1565 cells in total was carried out using the ImageJ Image Processing and Analysis tools.

**Differentiation of hPSC-derived limbal stem cells further towards corneal epithelium**

Cryopreserved p1 Regea08/017 hPSC-LSCs were thawed and 70 000 cells/cm^2^ were plated onto Ø13mm cell-culture treated plastic coverslips coated with Col IV/LN521. The cells were thereafter cultured to a confluent stage in CnT-30 (with pen/strep). After reaching confluence in a few days, the cells were acclimated to the future enriched medium conditions by supplementing the CnT-30 with 2.5% FBS and 0.5 mM CaCl_2_ for one day. In the following day, the cells were introduced to fully enriched differentiation conditions, which included transferring the cultures on top of mitotically inactivated 3T3-Swiss Albino mouse embryonic fibroblast feeder layers (CCL-92, ATCC, Manassas, VA) and changing the acclimation medium to fully enriched differentiation medium, CnT-30 (with pen/strep) supplemented with 5% FBS and 1 mM CaCl_2_ (both from Sigma-Aldrich).

Cells were thereafter cultured in enriched differentiation conditions and changing medium every day or every other day and replacing 3T3 feeder layers every 7 days. Fresh 3T3 feeder layers were prepared by thawing the Mitomycin C inactivated 3T3 feeders one day before, plating 25 000 cells/cm^2^ into 3T3 medium consisting of DMEM (with pen/strep), 10% FBS and 1X GlutaMAX™. During differentiation, morphology of the samples was monitored using a Nikon Eclipse TE2000-S phase contrast microscope. Cells were fixed and analyzed for their expression of CK3 and CK12 at 7-, 14- and 21-day time points.
