# Supplementary material for: Corneal epithelial differentiation of human pluripotent stem cells generates ABCB5+ and ∆Np63α+ cells with limbal cell characteristics and high wound healing capacity
Source: Stem Cell Res Ther. 2021 Dec 20;12:609. doi: 10.1186/s13287-021-02673-3 (PMC8691049; doi:10.1186/s13287-021-02673-3)
Supplement: Supplementary file 2 — Additional file 2: Containing supplementary data about CK3 and CK12 expression in post-thaw p1 hPSC-LSCs (Supplementary Fig. S1), ∆Np63α expression in hPSC-LSCs derived from the ABCG2+ cell population (Supplementary Fig. S2) and the Supplementary Movie S3 showing the wound closure of p1 hPSC-derived LSCs versus the tissue-derived porcine LSCs [file 13287_2021_2673_MOESM2_ESM.docx]

**SUPPLEMENTAL RESULTS**

**
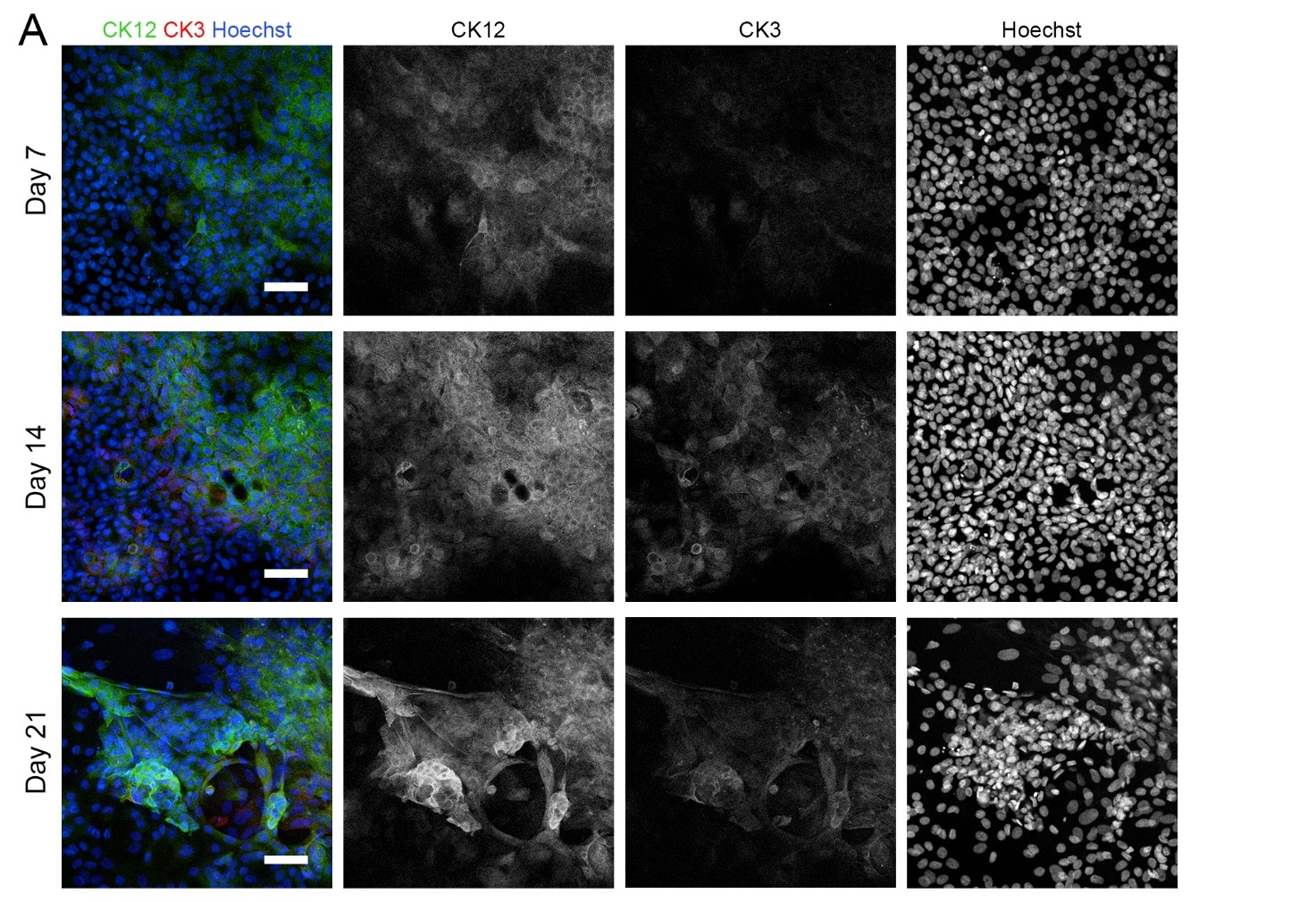
**

**Supplementary Figure S1. Cytokeratin 3 and 12 expression in hPSC-derived limbal stem cells/corneal epithelium.** Increasing expression of CK3 and 12 was observed during further differentiation of post-thaw p1 hPSC-LSCs in CnT-30 medium supplemented with 10% FBS and 1 mM CaCl_2_ after 7, 14 and 21 days on Collagen IV and laminin-521 coated coverslips in co-culture with mitotically inactivated 3T3 mouse embryonic fibroblast feeder cells. Scale bars, 50 µm. Data for line Regea08/017.


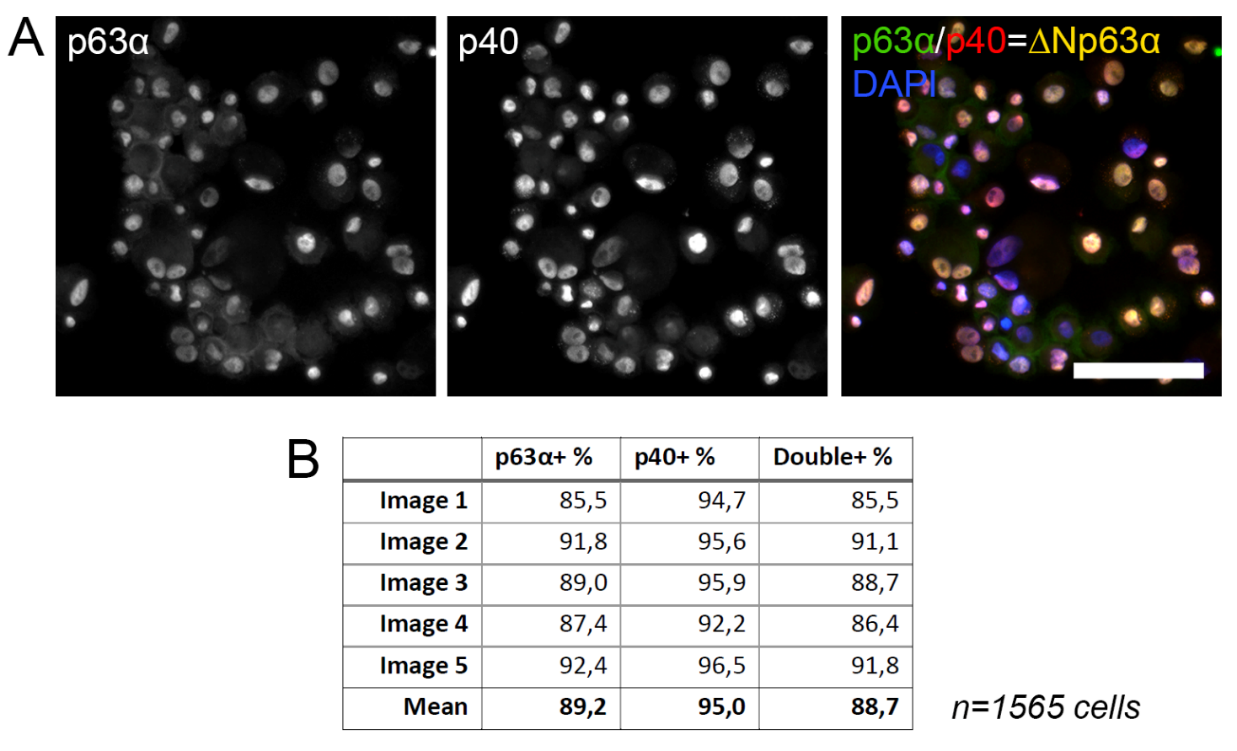


**Supplementary Figure S2. ∆Np63α expression in day 28 (day 11+17) hPSC-LSCs derived from isolated day 11 ABCG2^+^** **hPSC-LSCs. a** Representative immunofluorescence images of the cytospin sample stained with p63α an p40 antibodies. Cell nuclei counterstained with DAPI (blue). Scale bar, 100 µm. **b** Quantification of p63α, p40 and double-positive cells. Data for line Regea08/017.

**Supplementary Movie S3. Wound closure in hPSC-derived LSCs versus tissue-derived LSCs.** Representative time lapse recordings of parallel Regea08/017 p1 hPSC-LSCs (above) and p1 porcine LSCs (below) scratch assays. Frame interval 30 min, recording time 48 hours. Video presented in 8 frames per second.
